# α-Galactosylceramide Analogs with Weak Agonist Activity for Human iNKT Cells Define New Candidate Anti-Inflammatory Agents

**DOI:** 10.1371/journal.pone.0014374

**Published:** 2010-12-17

**Authors:** Gabriel Bricard, Manjunatha M. Venkataswamy, Karl O. A. Yu, Jin S. Im, Rachel M. Ndonye, Amy R. Howell, Natacha Veerapen, Petr A. Illarionov, Gurdyal S. Besra, Qian Li, Young-Tae Chang, Steven A. Porcelli

**Affiliations:** 1 Department of Microbiology and Immunology, Albert Einstein College of Medicine, Bronx, New York, United States of America; 2 Department of Medicine, Albert Einstein College of Medicine, Bronx, New York, United States of America; 3 Department of Chemistry, University of Connecticut, Storrs, Connecticut, United States of America; 4 School of Biosciences, University of Birmingham, Edgbaston, United Kingdom; 5 Department of Chemistry, National University of Singapore, Singapore Bioimaging Consortium, Agency for Science, Technology and Research (A*STAR), Biopolis, Singapore; New York University, United States of America

## Abstract

CD1d-restricted natural killer T cells with invariant T cell receptor α chains (iNKT cells) are a unique lymphocyte subset that responds to recognition of specific lipid and glycolipid antigens. They are conserved between mice and humans and exert various immunoregulatory functions through their rapid secretion of a variety of cytokines and secondary activation of dendritic cells, B cells and NK cells. In the current study, we analyzed the range of functional activation states of human iNKT cells using a library of novel analogs of α-galactosylceramide (αGalCer), the prototypical iNKT cell antigen. Measurement of cytokines secreted by human iNKT cell clones over a wide range of glycolipid concentrations revealed that iNKT cell ligands could be classified into functional groups, correlating with weak versus strong agonistic activity. The findings established a hierarchy for induction of different cytokines, with thresholds for secretion being consistently lowest for IL-13, higher for interferon-γ (IFNγ), and even higher for IL-4. These findings suggested that human iNKT cells can be intrinsically polarized to selective production of IL-13 by maintaining a low level of activation using weak agonists, whereas selective polarization to IL-4 production cannot be achieved through modulating the strength of the activating ligand. In addition, using a newly designed *in vitro* system to assess the ability of human iNKT cells to transactivate NK cells, we found that robust secondary induction of interferon-γ secretion by NK cells was associated with strong but not weak agonist ligands of iNKT cells. These results indicate that polarization of human iNKT cell responses to Th2-like or anti-inflammatory effects may best be achieved through selective induction of IL-13 and suggest potential discrepancies with findings from mouse models that may be important in designing iNKT cell-based therapies in humans.

## Introduction

Natural killer T cells (NKT cells) were originally defined as T cells that constitutively expressed NK-associated receptors in naïve mice [1]–[4]. Subsequent classification of subsets with these general properties has defined a major population known as type 1 or invariant NKT cells (iNKT cells), that express an invariant TCRα chain (Vα14Jα18 in mouse, Vα24Jα18 in human) which is paired with TCRβ chains of limited diversity. iNKT cells recognize lipids and glycolipids presented by the conserved non-polymorphic MHC class I-like molecule, CD1d, and recognize natural self or microbial glycolipids as well as a range of synthetic glycosylceramides [5]. The prototypic synthetic iNKT cell antigen is a synthetic α-galactosylceramide (αGalCer) known as KRN7000, which contains a C18 phytosphingosine linked with a saturated C26 N-acyl chain, has been extensively studied as a model antigen for iNKT cells in humans, as well as mice and other animal models, including rats and nonhuman primates [6]–[9].

Upon stimulation with KRN7000, iNKT cells exert multiple immuno-regulatory functions due in part to their rapid secretion of a wide range of cytokines. iNKT cells have a striking capacity to concurrently produce cytokines that are classically associated with both Th1 responses (e.g., IFNγ, TNFα) and Th2 responses (IL-4, IL-5, IL-13). Furthermore, their activation leads to induction of DC maturation, transactivation of NK cells and help to B cells [1]; [10]. Depending on the disease model considered, KRN7000-stimulated iNKT cells have shown an ability to modulate or improve immune responses in the context of tumors, microbial infections, allergic and autoimmune diseases [10]. An important parameter in the generation of iNKT cell-driven inflammatory responses is the ability of iNKT cells to stimulate DCs in a CD40L-dependent manner, which activates DCs to secrete IL-12 that can then stimulate NK cells to secrete IFNγ [11] or to exert tumoricidal activity [12]. Additional physiological functions of iNKT cells have been defined recently based on the fact that iNKT cells show a weak and CD1d-dependent reactivity to self lipid(s) (also referred to as “autoreactivity”). This is especially characterized by IL-13 and GM-CSF secretion, as shown most clearly for human iNKT cells upon coculture with monocytes [13] and DCs [14]. In the context of microbial infection, a higher degree of iNKT cell activation is achieved upon self-lipid recognition together with APC-derived IL-12 and IL-18, or type I Interferon co-stimulation, leading to a strong IFNγ production [13]; [15]; [16].

Because of the multiple immunological activities of iNKT cells, there have been intensive efforts recently to identify structural analogs of αGalCer that have the ability to selectively stimulate a limited range of iNKT cell functions. A particular emphasis has been on obtaining glycolipid agonists that stimulate a more restricted range of cytokine secretion compared to the mixed Th1 and Th2 type response that results from iNKT cell activation by KRN7000. These studies have provided several well characterized examples of variants of αGalCer that have the ability to skew mouse iNKT cell responses to either pure Th2 cytokine production and immunosuppressive activity or predominantly Th1 cytokine production and pro-inflammatory activity. Two prototypic Th2 polarizing agonists have been identified to date. These are an αGalCer analogue known as OCH, which contains a substantially truncated phytosphingosine chain (C9) and a slightly shorter N-acyl chain (C24) compared to KRN7000, and a potentially more potent Th2 skewing analog of αGalCer designated C20:2 because of its 20 carbon di-unsaturated N-acyl chain. These Th2 biasing analogs induce similar IL-4 production but much weaker and more transient IFNγ production when injected systemically into mice when compared to KRN7000. This appears to result mainly from a failure of these analogs to induce the transactivation of NK cells and their production of IFNγ as a secondary consequence of iNKT cell activation [11]; [17]. In contrast, the C-glycoside analog of KRN7000 was identified in mouse as Th1 polarizing compound that induces little IL-4 secretion but strong and sustained IFNγ production [18]. This compound does not induce detectable IL-4 production from mouse iNKT cells but leads to pronounced NK cell transactivation and IFNγ secretion [19]. Both OCH and the C20:2 analog have shown superior therapeutic effects compared to non-Th2-biasing activators such as KRN7000 when studied in various mouse models of autoimmune or inflammatory disease [20]–[23]. Similarly as predicted, the strongly Th1-biasing C-glycoside analogue has shown superior therapeutic effects in mouse models of cancer and chronic infection [18].

The mechanisms accounting for the cytokine biasing effects of αGalCer analogs such as OCH, C20:2 and C-glycoside are incompletely understood and remain a major focus for ongoing studies. Initial studies revealed that, in contrast to KRN7000, OCH weakly induced expression of c-rel, which is necessary for mouse iNKT cells to secrete IFNγ [24], and lower CD40L expression, IL-12 production and NK cell transactivation [11]. Recently, we demonstrated that, unlike KRN7000 which requires lysosomal loading onto CD1d, the C20:2 analog and other Th2 skewing analogs like OCH were characterized by a rapid and direct loading of cell surface CD1d proteins [17]. Another recent report showed that an active mechanism prevents Th2-polarizing analogs from being loaded in lysosomal compartments, thereby allowing them to be more selectively loaded at the cell surface [25]. This correlated with exclusion of the resulting CD1d/glycolipid complexes from detergent resistant microdomains of the APC plasma membrane [26]. Very recently, the ability of the C-glycoside to stimulate a Th1-polarized response following iNKT cell activation has been attributed to its more sustained presentation compared to KRN7000, despite the finding that iNKT cell TCRs showed a much lower binding avidity to complexes of C-glycoside bound to mCD1d [27].

To date, OCH has shown weak or no detectable ability to stimulate human iNKT cells [20]; [26]. Similarly, the C-glycoside analogue is only weakly stimulatory for human iNKT cells in culture [28]; [29]. In contrast, the C20:2 analogue is a strong agonist for human iNKT cells, and the cytokine secretion profile of cloned human iNKT cell lines is similar between C20:2 and KRN7000 [17]; [20]; [26]; [30]; [31]. These findings raise the question of whether analogs identified in the mouse model as selective activators of iNKT cell function will have the same or similar activities in humans, which is obviously an issue of major importance for attempts to develop agents of clinical utility. Given the above considerations and also several reports suggesting that subtle discrepancies in iNKT cell reactivity can be observed between mouse and human [17]; [20]; [26]; [30]–[33], there is a need for additional work comparing the quality of responses to iNKT cell antigens that stimulate responses in both mouse and human. In the current study, we have focused on the functional reactivities of human iNKT cells to KRN7000 and an extended panel of synthetic αGalCer analogs, including the previously identified cytokine polarizing compounds C20:2, OCH and C-glycoside, defined in the mouse system, as well as additional novel analogs. Analysis of the profile of cytokines secreted by cloned human iNKT cell lines over a wide range of glycolipid concentrations allowed the definition of strong and weak agonists. An important observation was that human iNKT cells secretion of IFNγ was induced at a lower degree of activation compared to induction of IL-4 secretion. Moreover, the design of a new *in vitro* assay, aiming to measure the ability of αGalCer-stimulated iNKT cells to transactivate other leukocytes, revealed that a strong iNKT cell agonist was necessary to induce NK cells to secrete IFNγ. Conversely, cytokine profiling indicated that weak glycolipid agonists had the best potential to induce a Th2-like quality of the direct iNKT cell response, because of the selective secretion of IL-13 and little or no IFNγ. These weak iNKT cell agonists may thus represent potential anti-inflammatory immunomodulators with potential applications in human iNKT cell-based therapy of inflammatory or autoimmune diseases.

## Materials and Methods

### Antibodies and staining reagents

For flow cytometric analysis, 6B11 (Vα24Jα18 specific) and mAbs specific for human CD3, CD4, CD8α, CD19, CD40L (CD154), CD56, CD69, GM-CSF & IFNγ were obtained from BD Biosciences. IL-4 and IL-13 specific mAbs were from Biolegend. Vα24 (C15) and Vβ11 (C21) specific mAb were from Immunotech. For antibody blocking in culture assays, non-specific isotype matched control, IL-2, IFNγ, IL-12p70 and CD40L specific mAbs without preservatives were obtained from BD Biosciences and IL-18 specific mAb from Medical & Biological Laboratories (MBL, Nagoya, Japan). Phycoerythrin and allophycocyanin labeled human CD1d tetramers were prepared in our laboratory as previously described [34]. Curves of tetramer equilibrium binding constants and graphs were plotted using the Graphpad Prism Software (Version 5). K_D_ values were calculated using the function non-linear fit (hyperbola, one binding site).

### Generation and cultivation of iNKT cell clones

The use of healthy donor PBMC (Peripheral Blood Mononuclear Cells) was reviewed and accepted by the Einstein Institutional Review Board. Written informed consent was obtained from all blood donors. CD3^+^Vα24^+^Vβ11^+^ were sorted from healthy donor PBMC using a MoFlo cell sorter (Beckman Coulter, Inc.) to deposit individual iNKT cells in wells of 96-well plates. Cells were expanded by PHA stimulation (PHA-P, Difco, Detroit, MI, which was reconstituted according to the supplier's instructions and used at a final dilution of 1∶2,000), in the presence of irradiated allogeneic PBMCs (3000 rad) and IL-2 at 250 IU/mL (Chiron). Culture medium was RPMI-1640 containing L-Glutamin (Gibco-BRL) supplemented with 10% fetal calf serum (FCS; Atlanta Biologicals) and 10 mM HEPES, 50 µM 2-mercaptoethanol, 1 mM Sodium Pyruvate and 1% nonessential amino acids mixture (all from Gibco-BRL). Cultures were maintained at 37°C in a humidified 5% CO_2_ incubator and were restimulated every 3 weeks and expanded by splitting and feeding with fresh medium with 250 IU/ml IL-2 as required. To avoid residual stimulation from PHA, the clones were tested in functional assays at least 2 weeks after the last PHA stimulation, when cells returned to a resting state, characterized by arrest of divisions and loss of their tear-drop shape. The TCR specificity of clones was validated by FACS using staining with mAbs specific for CD3, Vα24, Vβ11 and the invariant iNKT cell TCR chain (6B11), and the cell surface phenotype was determined by staining with CD4 and CD8α specific mAbs.

### Glycolipid preparation

All glycolipids used in this study were synthesized as previously described [17]; [34]–[36]. Glycolipids were initially dissolved in 100% DMSO at concentrations between 2 and 20 mM and then diluted to 500 µM in DMSO. Glycolipids dissolved in DMSO were stored frozen at -20°C. Immediately prior to use, the stock solutions were heated to 80°C for 10 minutes, followed by 10 minutes of sonication in a water bath sonicator (Branson model 2510, Fisher Scientific), and then mixed by high speed vortexing to ensure full dissolution of the glycolipids. For *in vitro* assays, the 500 µM glycolipid stocks in DMSO were diluted directly into prewarmed (37°C) culture medium with vigorous mixing and 10 minutes of sonication. This was followed by serial dilutions in 37°C medium with vigorous mixing.

### Cytokine ELISA

Capture ELISA assays were used for measurement of cytokine responses of human iNKT cells to αGalCer analogs. One day prior to the experiment, CD1d transfected HeLa cells [37] were plated at 25×10^3^ cells/well in 100 µL of medium in flat-bottom 96 well plates. Glycolipids were then added in 100 µL of medium to give final concentrations ranging from 0.05 to 10^4^ nM. The APCs were incubated for approximately 18 hours with the glycolipids and then irradiated (10,000 Rad) and washed twice with PBS prior to addition of 25×10^3^ iNKT cells well in 300 µL. Supernatants were harvested after 24 hours of co-culture and stored frozen at -20°C. After thawing, aliquots of the supernatants were assayed for specific cytokines by ELISA using capture and biotin-labeled detection antibody pairs specific for GM-CSF, IL-2, IL-4, IL-12p70 and IL-13 (all obtained from BD Biosciences), IFNγ (Thermo-Scientific) or IL-18 (MBL). Specific signals were developed using HRP- conjugated streptavidin (BD Biosciences) and Turbo TMB substrate (Thermo-Scientific). Supernatants were tested pure and/or diluted to give values within a quantitative linear standard curve generated with recombinant cytokines. Curves of dose-dependent cytokine responses and graphs were plotted using the Graphpad Prism Software. EC50 values were calculated using the function log (agonist) response with variable slope, and statistical analyses were carried out using the same software. To calculate concentration-dependent cytokine ratios, IL-4/IFNγ and IL-13/IFNγ ratios were calculated using cytokine values from which background levels (i.e. levels in cultures receiving DMSO vehicle alone) were subtracted. Ratios were calculated by dividing IL-4 or IL-13 concentrations in pg/mL by IFNγ concentration in ng/mL.

### In vitro transactivation assay and detection of CD40L expression

iNKT cells were stained for 5 minutes with 1–20 µM of CFSE (Molecular probes) and 5% FCS in PBS and washed twice. As a positive control, a fraction of CFSE-labeled iNKT cells were preactivated for 30 minutes with 1 µg/mL of PMA and 250 ng/mL of Ionomycin (Sigma-Aldrich) in culture medium and extensively washed. The experiment was initiated by adding CFSE-labeled unstimulated or PMA/Ionomycin-preactivated iNKT cells to freshly prepared PBMC pulsed with vehicle or αGalCer analogs at the indicated concentrations and at an iNKT/PBMC ratio of 1/10 or 1/40. Supernatants and cells were harvested from parallel conditions after the indicated time of co-culture. For intracellular cytokine staining, brefeldin A (Golgiplug, BD Biosciences) was added 4 h before harvesting the cells, which were fixed with 1% paraformaldehyde and permeabilized with 0.1% saponin (Sigma-Aldrich) prior to mAb staining. Experiments with blocking antibodies were performed with 10 µg/mL of preservative-free mAbs, including isotype matched control, anti-IL-2, anti-IL-12, anti-IFNγ and anti-CD40L (all from BD Biosciences) or anti-IL-18 (MBL). For detection of CD40L expression by iNKT cells, co-cultures of iNKT and PBMC were prepared as above, except that monensin (Golgistop, BD Biosciences) and fluorescent CD40L specific mAb (1/10 or 1/20 final dilution) were added at the beginning of the experiment, as previously described [38].

## Results

### Identification of strong and weak glycolipid agonists for human iNKT cells

**Figure 1:**
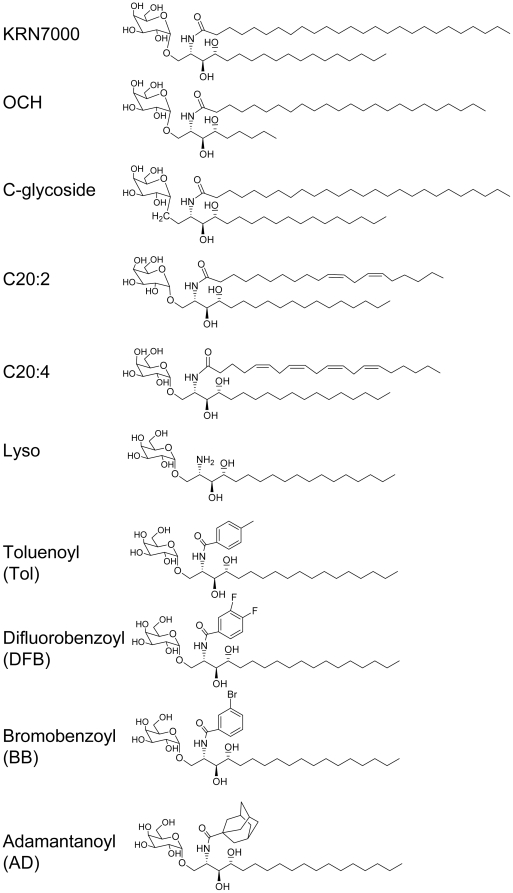
Structure of αGalCer analogs. Structures and nomenclature of synthetic αGalCer analogs used in this study. All analogs contain the same phytosphingosine core of KRN7000 with 18 carbons and 2S, 3S, 4R stereochemistry, except OCH which has a shorter phytosphingosine (C9) and shorter N-acyl chain (C24). The C-glycoside analog (C-Gly) is modified by a carbon linkage between the galactosyl residue and the ceramide moiety. Other structural analogs are modified with either no N-branching (Lyso), poly-unsatured N-acyl chains (C20:2 and C20:4) or with N-aromatic groups (Toluenoyl (Tol), Difluorobenzoyl (DFB), Bromobenzoyl (BB) or Adamantanoyl (AD)).

To characterize the quality of human iNKT cell responses to KRN7000 and to identify potential Th1 or Th2 skewing analogs, we assessed the reactivity of human iNKT cell clones to KRN7000 and multiple αGalCer analogs. The αGalCer analogs chosen for analysis included the previously described and characterized sphingosine chain truncated OCH compound [21], the C-glycoside variant of KRN7000 [18], and the Th2 cytokine biasing αGalCer C20:2 and C20:4 analogs [17]; [20]; [26] (Figure 1). In addition, a large panel of other less extensively characterized αGalCer analogs with a range of structural variations in their N-acyl chains were screened, and five novel αGalCer analogs were selected on the basis of their ability to stimulate *ex vivo* expansion of iNKT cells from PBMCs [35]. The analogs selected for detailed study included Lyso-αGalCer (with free amino instead of an amide linked acyl group on the phytosphingosine base) and αGalCer analogs with aromatic or adamantanoyl amide linked groups (Figure 1).

**Figure 2:**
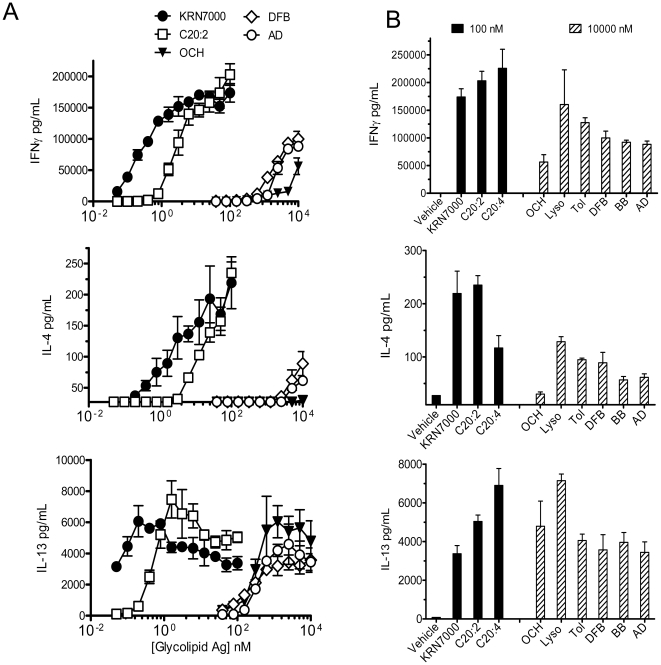
Cytokine responses of human iNKT cell clones to a dose range of αGalCer analogs. CD1d-transfected HeLa cells were incubated overnight with increasing concentrations of KRN7000 and αGalCer analogs (ranging from 0.05 to 100 nM for KRN7000, C20:2 and C20:4 for analogs; and 39 nM to 10 µM for other analogs; abbreviations for glycolipids as indicated in Figure 1) and then used as APC for iNKT cell stimulation. After 24 h of co-culture, supernatants were harvested, and cytokine levels were measured by ELISA. The sensitivity of detection was 27.4 pg/mL for IL-4 and 82.3 pg/mL for IL-13 and IFNγ. The data shown were generated with a CD4^+^ iNKT cell clone (HDD3) and are representative of another CD4^+^ clone (HDD11), as well as a double negative (DN, HDE3) and CD8α^+^ clone (HDA7). (A) Dose-response curves are presented for IFNγ, IL-4 and IL-13 (top to bottom panels respectively). Mean values measured from triplicate wells are indicated, and standard deviations are shown with brackets. Symbols for each glycolipid are indicated in the legend. No iNKT cell reactivity was detected with mock-transfected HeLa cells (not shown). (B) HDD3 iNKT cell clone cytokine responses measured for all glycolipids using 100 nM of KRN7000, C20:2 and C20:4 (black bars) or 10000 nM (10 µM) of other analogs (cross hatched bars). This clone did not display detectable reactivity to the C-glycoside (Supplementary Figure S2).

As previously observed, some iNKT cell clones displayed variable reactivity to CD1d-transfected APCs in the absence of exogenously added synthetic antigen, which manifested as significant secretion of IL-13 and GM-CSF and little or no IFNγ production (Supplementary Figure S1). This pattern of “autoreactivity” was CD1d-dependent, since the same iNKT cells did not react to mock-transfected HeLa cells (not shown), and suggested that iNKT cells can recognize lipid(s) derived from HeLa APC. The assessment of iNKT cell reactivity to a wide range of concentrations of KRN7000 (0.05 nM to 100 nM) revealed that the most sensitive indicator of an Ag-dependent response of iNKT cells among the cytokines measured was IL-13 production, which was induced at very low KRN7000 concentration (≤0.1 nM), and reached maximal production at a concentration of approximately 1 nM (Figure 2). IFNγ was induced with a similar profile, but required about a 1 log higher concentration of KRN7000 to be induced and to reach a plateau response. Interestingly, IL-4 production showed the highest threshold concentration of KRN7000 for induction of detectable secretion, and induction of this response was saturated only at very high KRN7000 concentrations in the µM range (not shown). In addition, the maximal levels of secreted cytokines in pg/mL were lower for IL-4 compared to IL-13, and even lower compared to IFNγ.

Responses of iNKT cell clones to the C20:2 analogue were associated with similar or slightly weaker dose-dependent cytokine profiles compared to KRN7000 (Figure 2A and not shown), indicating a potency or an agonistic activity comparable to KRN7000, as previously suggested [20]; [26]; [30]; [31]. A substantially lower iNKT cell response was observed with the C20:4 analog, as well as with the lyso- and toluenoyl- αGalCer analogs (Figure 2B). All iNKT cell clones tested displayed a much weaker reactivity to OCH, and relatively high concentrations (>10 nM) of this analog were required to induce detectable IL-13 production or weak IFNγ secretion (Figures 2A and 2B). OCH mediated-induction of IL-4 was minimal with all clones tested. A pattern similar to that of OCH was observed with the N-aromatic branched analogs. The data shown in Figure 2 were generated with a CD4^+^ iNKT cell clone (HDD3) and are representative of another CD4^+^ clone (HDD11), as well as a double negative (DN, HDE3) and CD8α^+^ clone (HDA7). Large cytokine titrations for the DN clone and the CD8α^+^ clones are presented in supplementary Figure S2 and S3, respectively. The other clones tested displayed a comparable sigmoidal response to analogs, with a similar strong agonist activity for KRN7000 or the C20:2 analog; and a weak agonist activity for the remaining analogs. However, the clones differed in their degree of “autoreactivity” (Supplementary Figure S1) and in the magnitude of cytokines they were able to secrete, i.e. the DN or the CD8α^+^ clones systematically produced maximal amounts that were lower than the amounts produced by the CD4+ clones (Figure2, Supplementary Figure S2/S3 and data not shown).

Surprisingly, only a fraction of the iNKT cell clones tested displayed a weak, but detectable, reactivity to the C–glycoside analog, which was comparable to the response to OCH, with primarily IL-13 production and a limited induction of IFNγ (Supplementary Figure S4) and data not shown). Two clones out of six clones tested displayed a detectable reactivity to the C–glycoside analog. Altogether, the patterns of cytokine secretion observed over a large range of antigen concentrations suggested that iNKT cell agonists have variable potency or agonistic ability, with some analogs being strong agonists while other are intermediate or weak agonists.

CD1d-transfected Hela cells might not be representative of the presentation by DCs, so we performed a comparative analysis, with more limited analog concentrations, between Hela-CD1d cells and monocyte-derived DC (Supplementary Figure S5). Comparable cytokine profiles were observed between Hela-CD1d cells and DC, with IL-13 being induced at the lowest Ag stimulation and with KRN7000 and C20:2 analogs being strong agonists while other behaved as intermediate or weak agonists. However we observed a higher cytokine levels produced upon stimulation with Hela cells, which can be explained by a higher CD1d expression.

### Characterization of strong versus weak iNKT cell agonists using CD1d tetramers

**Figure 3:**
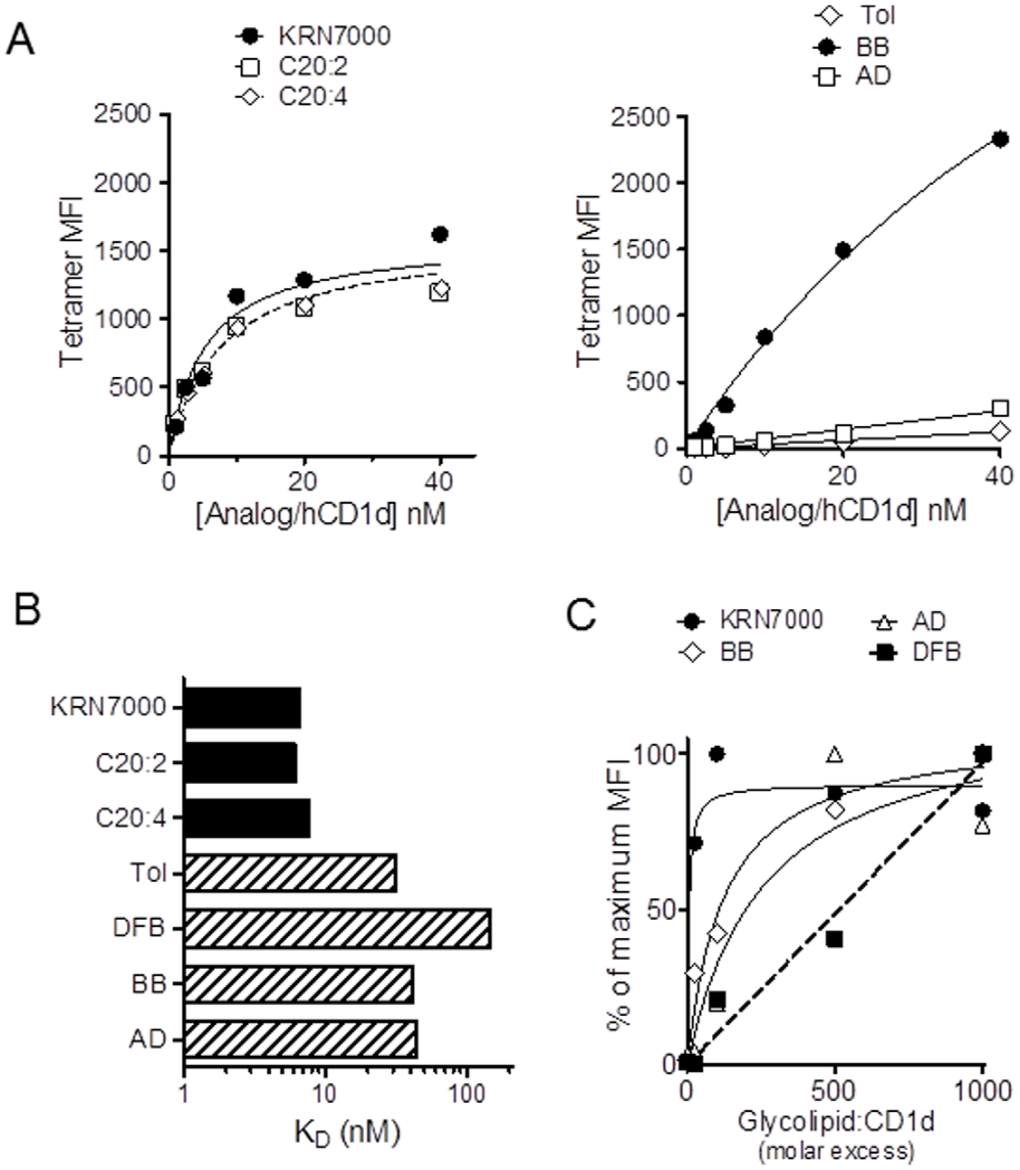
Characterization of human iNKT cell TCR interactions with analog-loaded tetramers. (A) iNKT cell clones were stained with titrated amounts of hCD1d tetramers loaded with the indicated glycolipids (abbreviations as in legend to Figure 1), and the measured MFI values are plotted for selected glycolipids. (B) The equilibrium dissociation constants (K_D_, inversely proportional to TCR avidity) were determined as the concentrations of tetramers required to yield 50% of maximal binding. Data shown were obtained using the CD4^+^ iNKT cell clone HDD3, and similar results (not shown) were obtained with a second CD4^+^ clone (HDD11) and a CD4^−^8^−^ double negative iNKT cell clone (HDE3). (C) Analysis of iNKT cell clones labeled with 20 nM of hCD1d tetramers which were loaded with variable excesses of analog to constant hCD1d (25 to 1000 fold molar excess of glycolipid to hCD1d protein). The measured MFI values were normalized as the percent of the maximum staining intensity obtained with each analog loaded tetramer. Data presented were generated with HDD3 (CD4+) iNKT cell clone, and similar results (not shown) were obtained with HDE3 (DN) clone.

To characterize in more detail the iNKT cell TCR recognition of αGalCer analogs presented by human CD1d, we generated soluble and fluorescent tetrameric hCD1d molecules loaded with each of the glycolipid analogs. Staining of iNKT cell clones showed that tetramers could be reliably generated with all compounds, with the exception of tetramers loaded with the lyso-αGalCer analog, which failed to detectably stain any of our iNKT cell clones. This was similar to the previously reported failure of mouse CD1d tetramer loaded with lysosulfatide to bind detectably to murine non-invariant and sulfatide specific NKT cells, despite a clear reactivity to this lipid upon presentation by APCs [39]; [40]. Tetramers loaded with OCH and C-glycoside provided only a weak staining intensity of a subset of iNKT cell clones tested (Supplementary Figure S2 and not shown), as we previously observed [17]; [20]. To quantitate their binding avidities to iNKT cell TCRs, we used titration of analog-loaded hCD1d tetramers to determine equilibrium dissociation constants (K_D_), which are inversely proportional to avidity (Figure 3A and 3B). Human iNKT TCRs displayed a strong avidity for KRN7000, C20:2 and C20:4 analogs in complex with hCD1d, as previously described [17]; [20]; [26], with K_D_ values in the 2–5 nM range. However, all analogs with N-aromatic branching displayed a weaker avidity, as they generated lower intensities of tetramer staining and higher K_D_, in the 30–80 nM range (Figure 3A and B), consistent with functional studies identifying them as weak iNKT cell agonists compared to KRN7000. In addition, when tetramers were loaded using a range of glycolipid to CD1d ratios, we found that tetramers were efficiently loaded at a 25–50 fold lipid excess for KRN7000 and for toluenoyl-, bromobenzoyl- and adamantanoyl-derivatives of αGalCer. However, a much higher lipid excess was necessary to achieve optimal loading with difluorobenzoyl analog (Figure 3C). These findings suggested that all of the N-aromatic branched compounds studied had weak iNKT TCR avidity, and the difluorobenzoyl analog also had weak binding to hCD1d.

**Figure 4:**
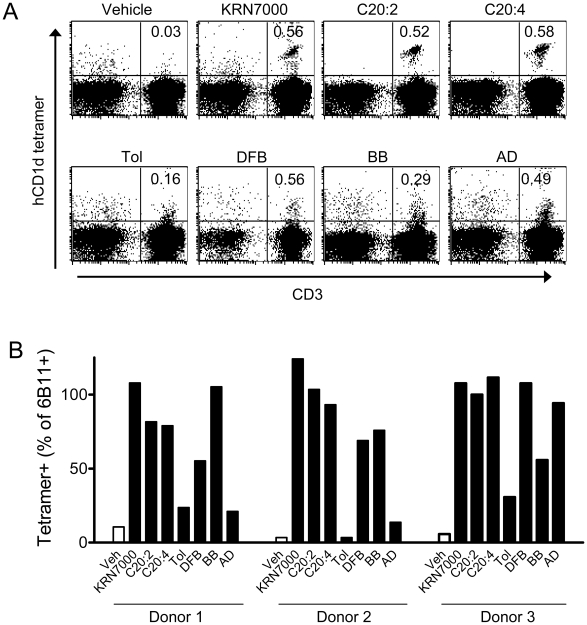
*Ex vivo* analysis of PBMC with CD1d tetramers loaded with αGalCer analogs. (A) Flow cytometric analyses were performed after labeling of PBMCs from healthy donors with hCD1d tetramers loaded with each of the indicated glycolipids. Representative dot plots generated with PBMCs from one normal donor, with the analog tested indicated at the top of each plot and the percentages of tetramer^+^ CD3^+^ cells indicated in the top right quadrants. The dot plots show events gating as live lymphocytes (PI negative and FSC/SSC gated lymphocytes). (B) Comparison of tetramer^+^ cell frequencies in PBMC from three different donors. The frequencies of cells specifically stained with hCD1d tetramers loaded with the indicated glycolipids (or vehicle (Veh) only (i.e., DMSO)) are shown as the percentage of total iNKT cells (i.e., cells staining with mAb 6B11^+^, specific for the invariant TCRα chain of iNKT cells). Similar results were obtained for three additional PBMC donors (not shown).

Our panel of clones might be limited to iNKT cells able to expand upon PHA stimulation and might be representative of a fraction only of the iNKT cell repertoire. To demonstrate that the iNKT cell cross-reactivity between KRN7000 and αGalCer analogs was not limited to the clones tested, but valid for the majority of iNKT cells, we performed *ex vivo* tetramer staining of PBMCs from healthy donors with variable iNKT cell frequencies (Figure 4A). Comparable iNKT cell frequencies were observed between staining with Vα24Jα18 specific Ab 6B11 and KRN7000-, C20:2- and C20:4-loaded tetramers (Figure 4B). The frequencies of iNKT cells measured using tetramers loaded with N-aromatic branched analogs were lower in some or all PBMCs tested. A lower detection might be explained by weaker efficiency of staining, since lower MFI values were also seen with iNKT cell clones stained with tetramers loaded with these glycolipids. However, tetramer staining of PBMCs suggested that at least a substantial fraction of the iNKT cell repertoire was able to bind tetrameric CD1d complexes loaded with weak agonist glycolipids with N-aromatic branching.

### Hierarchy of anti-inflammatory versus pro-inflammatory iNKT cell cytokine induction

**Figure 5:**
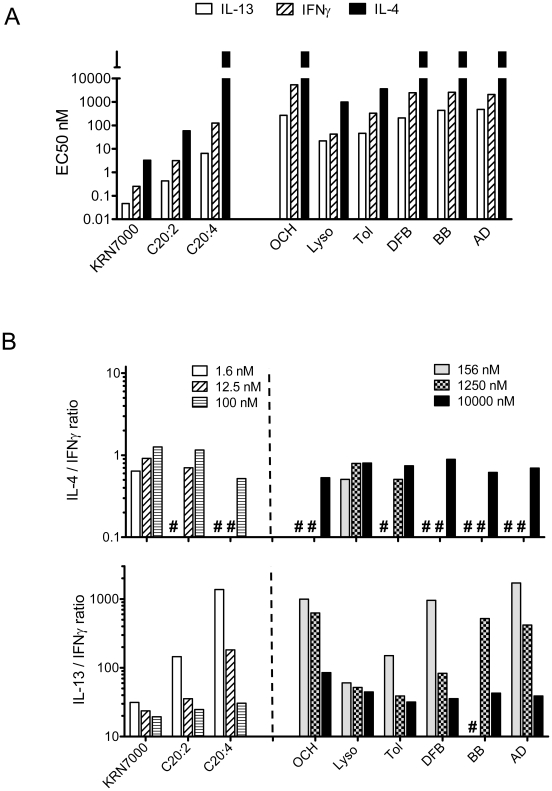
Antigen and dose-dependent cytokine polarization of human iNKT cells. (A) EC50 values (Ag concentration required to obtain half maximal cytokine response) were calculated for production of the indicated cytokines in response to each of the glycolipid agonists. Values are presented on a logarithmic scale, and EC50 values of IL-4 for C20:4, OCH, DFB, BB and AD compounds could not be calculated because IL-4 levels did not reach a plateau and were >10 µM. (B) To show variations in cytokine ratios depending on the analog concentration tested, IL-13/IFNγ ratios and IL-4/IFNγ ratios are presented on the bottom and top panel, respectively (calculated as explained in Materials and Methods). Note that ratios could be calculated only when IL-4 and/or IFNγ were detectable and the # symbol indicates ratios which could not be calculated. Concentrations of antigens are indicated as in figure 2A, with low concentrations tested for KRN7000, C20:2 and C20:4 (1.6, 12.5 and 100 nM, white background bars) and high concentrations of other analogs (156, 1250 and 10000 nM, gray background bars). Data presented in this figure were interpreted from results shown in Figure 2, and IL-13/IFNγ & IL-4/IFNγ ratios calculated for HDD3 and HDE3 clones with DCs are presented in supplementary Figure S6.

A strikingly consistent feature of our analysis of the responses to various αGalCer analogs was the induction of IL-13 secretion at a lower degree of activation when compared to induction of the other cytokines tested. This was readily observed by examining EC50 values of the various ligands for production of each cytokine (Figure 5A). Indeed, regardless of the potency of the glycolipid, the EC50 values for IL-13 were in all cases lower than the EC50 values for IFNγ, and even lower than those for IL-4 (Figure 5A). This suggested that human iNKT cells do not polarize in an antigen-dependent manner, but more likely do so in a manner that depends on their degree of activation. This was reflected by dose-dependent variations in IL-13/IFNγ ratios which indicated a more pronounced Th2-type bias at lower glycolipid concentrations, which was not evident for IL-4/IFNγ ratios which appeared more constant (Figure 5B). Altogether, these data suggested that human iNKT cells can be polarized to IL-13 production with a weak stimulus, whereas a true polarization to IL-4 without IFNγ production may not be possible. Similar patterns of high IL-13/IFNγ ratios at low activation degrees and stable IL-4/IFNγ ratios were also observed with DCs (Supplementary Figure S6).

Interestingly, iNKT cells with autoreactivity showed high IL-13/IFNγ ratios in absence of synthetic antigen (supplementary Figure S1 and data not shown), and the highest IL-13/IFNγ ratios were found with a low degree of Ag-dependent activation for all clones (i.e., at suboptimal doses of agonists) (Figure 5B and Supplementary Figure S6). Thus, the responses of human iNKT cell clones induced with weak agonists, or suboptimal doses of strong agonists, was comparable to the pattern of activation previously reported in the context of “autoreactivity” in response to self lipids, especially with regard to the predominant expression of IL-13 and GM-CSF [13]. Since full activation of iNKT cells has been reported to occur in the context of autoreactivity and costimulation with IL-12 plus IL-18 [13]; [15], we examined the effect of these two cytokines on the responses to weak agonist glycolipids or suboptimal doses of strong agonists. This showed that addition of IL-12 plus IL-18 markedly enhanced the IFNγ secretion from such cultures. In contrast, IL-13 secretion was generally reduced under these conditions, while IL-4 and GM-CSF secretion were only modestly increased or not affected (Supplementary Figure S7). Altogether, these findings suggested that weak suboptimal stimulation with weak αGalCer agonists induced a pattern of cytokine secretion that was similar to that associated with the iNKT cell response to self lipid(s).

### An *in vitro* system for assessment of NK cell transactivation

Studies of mouse iNKT cell responses have indicated that the strong IFNγ production resulting from stimulation with KRN7000 and other strong iNKT cell agonists originates from the secondary activation of NK cells to secrete IFNγ [11]; [17]; [19]; [41], in addition to iNKT cell –derived IFNγ. This suggested that a significant component of the cytokine polarization of responses to iNKT cell activating glycolipids *in vivo* depends on the extent to which IFNγ and potentially other proinflammatory cytokines are produced by other leukocytes that undergo transactivation downstream of the initial iNKT cell stimulation. To model this important component of the overall response for human iNKT cell responses, we developed an *in vitro* assay to assess the ability of human iNKT cells to transactivate NK cells for IFNγ production.

**Figure 6:**
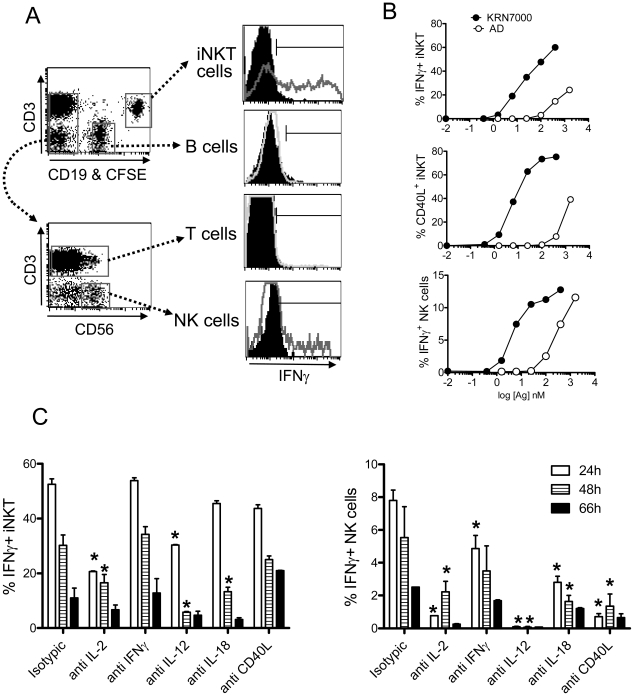
A novel *in vitro* “transactivation assay” to assess the ability of human iNKT cells to activate other leukocytes. (A) Format for analysis of transactivation of bystander leukocytes. Flow cytometric analysis was carried out for culture containing iNKT cells co-cultured for 24 hours with fresh human PBMC at a 1/10 ratio. For the intracellular detection of IFNγ, Brefeldin A was added for the last 4 hours. Representative electronic gating is shown for the discrimination of CFSE-labeled iNKT cells, B cells, T cells, and NK cells to measure IFNγ expression (or CD69 expression, not shown). Filled black histograms show staining with vehicle control and are overlaid with open gray histograms for stimulation with 100 nM of KRN7000. (B) Dose-dependent IFNγ and CD40L expression by iNKT cells (top and middle graph, respectively) and IFNγ production by transactivated NK cells (bottom graph), after 24 h of co-culture of CFSE-labeled iNKT cells and PBMCs at a 1/10 ratios and the indicated concentration of either KRN7000 (black filled circles) or the adamantanoyl (AD) analog (open circles). Data are presented for a CD4^+^ clone (HDD3), and similar results (not shown) were obtained with a DN clone (HDE3). (C) Effect of specific blockade of various cytokines and CD40L on the activation of iNKT cells and transactivated NK cells. CFSE-labeled iNKT cells and PBMCs were co-cultured at a 1/40 ratio for the indicated time, in the presence of KRN7000 at 25 nM and 10 µg/mL of isotype matched control or specific blocking antibodies as indicated. Average percentages of IFNγ^+^ iNKT cells and NK cells, assessed by intracellular staining at 24, 48 and 66 hours, are indicated in the left and right plots, respectively. Values significantly decreased as compared to isotypic control are indicated with asterisks (P<0.05 in 2-way ANOVA).

While mouse iNKT cells activated with KRN7000 are well documented to induce IFNγ production by NK cells [11]; [17]; [19]; [41], this has not previously been shown to be the case with human iNKT cells. Thus, although augmentation of NK cell cytolytic activity has been observed in human cell cultures following iNKT cell stimulation with KRN7000 [42]–[44], NK cell cytokine production has not to our knowledge been studied. Using fresh human PBMCs co-cultured with αGalCer analogs and CFSE-labelled iNKT cells, we were able to discriminate iNKT cells from other cells in flow cytometric analysis without using TCR-specific Abs or αGalCer-loaded CD1d tetramers, which might fail to detect them due to TCR downregulation (Figure 6A). The PBMCs were used as a source of physiological CD1d^+^ APC (i.e. monocytes, B cells and rare DCs), as well as a source of T, B and NK cells which, can be potentially transactivated by iNKT cells.

Preliminary experiments revealed that a detectable human NK cell transactivation, monitored by intracellular IFNγ staining, could be induced by iNKT cells pre-activated with PMA/Ionomycin. Peak levels of NK cell transactivation were observed after 20–24 h, with an iNKT/PBMC ratio of 1/10 to 1/40 (not shown). Furthermore, upon iNKT cell stimulation by KRN7000, nearly all B cells and NK cells, but not T cells, became CD69^+^. A similar high degree of CD69 induction in B and NK cells was also observed with the C20:2 analog, while iNKT cell stimulation with analogs of weak potency induced intermediate levels of CD69 positivity in B and NK cells (not shown). Staining for intracellular IFNγ showed that iNKT cells already had substantial expression of IFNγ at 6 h, and this continued to increase at 24 h (not shown). Production of IFNγ was detectable at 24 h in NK cells, but not in T cells or B cells (Figure 6A). Thus, this *in vitro* system allowed us to observe that, in a manner comparable to what has been described *in vivo* in mice [11]; [17]; [19]; [41], KRN7000-activated human iNKT cells responded rapidly and induced a subsequent transactivation of NK cells. This transactivation was characterized here by IFNγ induction, and fits with the previously reported ability to induce cytolytic activity [42]–[44]. Preliminary studies of intracellular cytokine expression revealed that iNKT cells were the major producers of IL-13, IL-4 and GM-CSF in this co-culture system (not shown), and these cytokines were not detected by intracellular staining in transactivated cells.

### Factors controlling NK cell transactivation by human iNKT cells

Using this *in vitro* system, we examined some of the potential factors that may lead to NK cell transactivation. Previous investigations in mice suggested that NK cells are activated to secrete IFNγ by IL-12 released from APC in response to CD40L expression by iNKT cells [11], with a significant contribution also attributable to iNKT cell-derived IFNγ [41]. In addition, it was recently described that mouse NK cells require IL-18 sensitization to become responsive to IL-12 [45], and studies with human iNKT cells have implicated IL-2 and IFNγ in the stimulation of the cytolytic activity of NK cells [42]–[44]. However, the role of iNKT cell-derived IL-2 and IFNγ to stimulate NK cell secretion of IFNγ has not been determined. We therefore tested whether IL-2, IFNγ, CD40L, IL-12 and IL-18 were involved in the process of NK cell transactivation as a result of iNKT cell activation, in our *in vitro* system. We observed a dose-dependent upregulation of CD40L by iNKT cells (Figure 6B) which correlated with the level of iNKT cell activation (assessed by intracellular IFNγ staining). This appeared to correlate with the agonistic potential of the analog tested, as CD40L and IFNγ were also induced with high doses of the weak adamantanoyl agonist (Figure 6B). The degree of NK cell transactivation correlated well with both the degree of CD40L expression by iNKT cells and the degree of IFNγ secretion by iNKT cells, suggesting that CD40L and IFNγ expression by iNKT cells indeed contributed to NK cell transactivation. Moreover, the specific blockade of IL-2, IFNγ, CD40L, IL-12 or IL-18 with mAbs decreased the NK cell transactivation mediated by KRN7000 stimulation (Figure 6C).

While blocking of IFNγ and CD40L had no effect on the activation iNKT cell, either IL-2, IL-12 or IL-18 blockade decreased the duration of iNKT cell production of IFNγ after 48 h. This suggested that iNKT cells are sustained by a “feedback” loop involving IL-12 and IL-18, presumably derived from APCs, and IL-2 in an autocrine manner. Also IL-13 levels detected in supernatants appeared to be decreased upon IL-2 and IL-18 blocking (not shown). Interestingly, the specific blocking of any factor tested led to a detectable decrease of NK cell transactivation (Figure 6C). Whereas neutralization of IFNγ, IL-2, CD40L and IL-18 showed a partial blocking effect, IL-12 blockade completely abolished the NK cell transactivation. These blocking effects translated to a decrease in the overall IFNγ secreted in supernatants (not shown).

### Failure of weak iNKT cell agonists to induce NK cell transactivation

**Figure 7:**
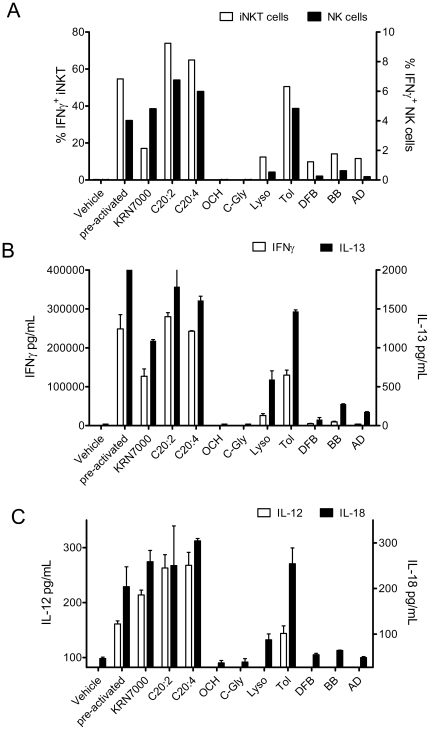
Comparison of αGalCer analogs for their ability to induce NK cell transactivation. (A) iNKT cell clones were co-cultured for 24 hours with fresh human PBMC at a 1/10 ratio with 100 nM of the indicated analogs, and Brefeldin A was added for the last 4 hours. The percentages of IFNγ^+^ iNKT cells (open bars) and IFNγ^+^ NK cells (filled bars) are indicated. Cytokine levels were assessed by ELISA in supernatants from identical conditions in absence of Brefeldin A treatment: (B) IFNγ levels (open bars) and IL-13 levels (filled bars); the detection limit was 82.3 ng/mL for both cytokines. (C) IL-12 levels (open bars) and IL-18 levels (filled bars); the detection limit was 27.4 ng/mL. Results obtained with a CD4^+^ clone (HDD3) are presented, and similar data (not shown) were obtained with a DN clone (HDE3).

Comparison of αGalCer analogs with KRN7000 in the transactivation assay at a fixed concentration (100 nM) showed that the C20:2, C20:4 and toluenoyl analogs had a similar or even higher ability to activate iNKT cells and stimulate NK cell transactivation (Figure 7A). This translated into a high global production of IFNγ in association with high IL-13 levels. This also correlated with detectable IL-12 generation and enhanced IL-18 levels compared to vehicle control. Multiple other αGalCer analogs, with 1 or 2 unsaturations in the N-acyl chain, also displayed a comparable pattern (not shown). On the other hand, weak agonists, such as the lyso, difluorobenzoyl, bromobenzoyl and adamantanoyl analogs, induced a lower degree of iNKT cell activation with a limited transactivation of NK cells. This translated into lower global IFNγ production and lower, but still detectable, IL-13 levels, while IL-12 was undetectable and IL-18 levels remained low. Altogether, the ability of iNKT cells to transactivate NK cells appeared to correlate with the agonistic potency of αGalCer analogs, with agonists of strong or intermediate potency generating subsequent NK cell transactivation, while weak agonists partially activated iNKT cells and led to limited or no NK cell transactivation.

## Discussion

In the current study, the analysis *in vitro* of human iNKT cell responses to cytokine polarizing αGalCer analogs defined in mouse (e.g. OCH, C20:2 and C-glycoside) and the identification of new analogs with N-aromatic branching have allowed us to define different states of human iNKT cell activation. The lowest detectable activation state of human iNKT cells was characterized by IL-13 and GM-CSF secretion, as previously observed in studies of the autoreactivity of these T cells against DCs and monocytes [13]; [14]; [46] or in response to CD1d transfected cells (this work and [13]; [31]; [47]; [48]. Using CD1d-transfected Hela cells and dendritic cells as APCs, this pattern could be induced by using either suboptimal doses (<1 nM) of strong agonists such as KRN7000 or the C20:2 analog, or by using higher doses of weak agonists with N-aromatic branching (∼100 nM). Importantly, this was achieved in cultures of cloned iNKT cells which did not allow the recruitment of responses resulting from the transactivation of NK cells. These results indicate that it is possible to manipulate human iNKT cell responses using synthetic glycolipids in a manner that mimics or even enhances the response to self-derived natural antigens presented by CD1d^+^ APCs. This function might correspond to a physiological “steady state” or default regulatory function, in absence of direct TCR recognition of microbial or synthetic glycolipids presented by CD1d and in absence of IL-12 and IL-18 or type I IFN co-stimulation provided by activated APCs [13]; [15].

In contrast, a distinct and apparently higher state of iNKT cell activation was achieved by using either standard doses of strong agonists (∼100 nM) or high doses of weak agonists (i.e. >400 nM). This was characterized by maximal IL-13 production by human iNKT cells, along with high production of IFNγ and induction of IL-4 secretion. Importantly, this correlated with transactivation of NK cells which contributed to a much higher global IFNγ secretion, and thereby biasing to a more Th1 polarized response. The distinction between a low versus high degree of iNKT cell activation might rely on the ability of analogs to form stable glycolipid/CD1d complexes and also on the affinity of the iNKT cell TCR for these glycolipid/CD1d complexes [31]. These properties would lead to long lasting iNKT/APC interaction [13]; [31], resulting in augmented intracellular calcium signaling and ERK phosphorylation [13]. Such enhanced TCR signaling in iNKT cells appears to be required for their secretion of IFNγ, IL-4 and IL-2 secretion from human iNKT cells [13] and possibly also for the secondary transactivation of NK cells based on findings from our study.

Our results obtained using human CD1d tetramers to measure the avidity of equilibrium binding (K_D_), as well as studies of tetramer binding kinetics, suggested that the biasing of iNKT cell cytokine responses to selective IL-13 secretion observed with the N-aromatic branching analogs of αGalCer can be explained by low iNKT cell TCR avidity. However this does not exclude the possibility that other mechanisms may also contribute to the nature of the iNKT cell response that is triggered by these compounds. For example, these weak analogs might be preferentially loaded at the cell surface rather than at intracellular sites, and they might be excluded from CD1d molecules positioned in detergent-resistant plasma membrane microdomains, both of which are mechanisms that we have previously identified to alter the quality of the iNKT cell responses in mice [17]; [26]. Additional studies will be required to test this possibility. It is important to note that results from the current study clearly imply that IFNγ production by human iNKT cells occurred at a lower threshold of activation than IL-4 production, indicating that a selective polarization of iNKT cells to IL-4 production without IFNγ secretion might not be possible for human iNKT cells. This contrasts with previous findings from one study of mouse iNKT cells, for which IFNγ production was reported to require a stronger TCR activation signal than IL-4 as a result of a signaling mechanism involving c-rel expression [24]. Although this may represent a significant functional difference between mouse and human iNKT cells, our data suggest that it may still be possible to effectively polarize human iNKT cells to a “Th2-like profile” characterized by IL-13 production, instead of IL-4 production.

The preferential production of IL-13 by human iNKT cells activated with weak agonists may have important implications in the therapeutic manipulation of these cells for treatment of autoimmunity or other inflammatory conditions. Since the range of immunologic effects of IL13 may not overlap completely with those of IL-4 [49], it will be critical to determine the potential benefit provided by responses biased toward IL-13 production in the context of autoimmune disease. For example, since IL-13 might generate adverse effects, such as induction of allergy or asthma [50], and a profibrotic effect of IL-13 secreted by hepatic iNKT cells has been suggested in the context of chronic HCV infection [51]. On the other hand, additional studies are necessary to determine whether IL-13 might stimulate potentially beneficial immunosuppressive activity by inducing alternatively activated (M2) macrophages with anti-inflammatory properties [52]; [53] or by inducing TGFβ production from immature myeloid cells, as was described for responses of non-invariant NKT cells in tumor bearing mice [54]; [55].

In the current study we also describe a new *in vitro* assay to determine the ability of human iNKT cells activated by glycolipid antigens to transactivate other leukocytes, especially induction of IFNγ production by NK cells, which can contribute in a major way to IFNγ in the setting of iNKT cell responses in mice [17]; [19]; [41]. Our data suggest that human NK cells can effectively be transactivated by human iNKT cells in a similar manner to that described in mouse and that this is correlated with a high degree of iNKT cell activation. Consistent with previous mouse data, this process involved CD40L upregulation and secretion of IFNγ and IL-2 by iNKT cells, as well as IL-12 and IL-18, probably derived from APC upon CD40 signaling [56]. While human iNKT cell-derived IL-2/IFNγ are known to induce cytolytic activity from NK cells [42]–[44], we show here that these cytokines are also involved in the induction of IFNγ secretion by human NK cells. To our knowledge, we describe for the first time the ability of activated iNKT cells to induce IL-18 release and suggest that human NK cells might require sensitization by IL-18 to respond to IL-12 in the manner that has been documented for mouse NK cells [45]. In addition, the induction of IL-12 and IL-18 in our system appeared to sustain iNKT cell expression of IFNγ and fits with the previously described responsiveness of iNKT cells to these cytokines [13]; [15]; [16]; [57].

Further validation of our *in vitro* transactivation assay will require comparison with *in vivo* data generated in non-human primates or in human clinical studies. This is required to determine the physiological relevance this new assay. To date the activity of KRN7000 in humans has been tested only in cancer patients, who are known to display numerical and/or functional iNKT cell defects [12]. To our knowledge, KRN7000 and its structural analogs have never been tested in healthy humans. More effort is thus needed to better evaluate iNKT cell responses in experimental systems that may adequately model human immune responses *in vivo*. For example, this could involve testing the effects of analogs in non-human primates or in humanized mouse models (e.g., mice engineered to express human CD1d and adoptively transferred with human iNKT cells). Importantly, our data are suggestive that the clinical use of polarizing analogs should consider the possibility that some analogs might induce distinct, species-dependent reactivity, as previously observed with synthetic antigens or microbial analogs [17]; [20]; [28]–[30]; [32]; [33]. We here confirmed that human iNKT cells display a low reactivity *in vitro* to OCH [17]; [20] and C-glycoside [28]; [29], and our novel transactivation assay unexpectedly suggested that the C20:2 analog can induce a KRN7000-like profile, at least *in vitro.* These findings are in general agreement with most previously reported studies on the *in vitro* reactivity of human iNKT cell clones to various αGalCer analogs [20]; [26]; [30]; [31].

A number of published observations have suggested that significant differences may exist between mouse and human iNKT cells in terms of their responses to synthetic and natural glycolipid antigens. Human and mouse CD1d proteins have been shown to have distinct abilities to bind or present certain glycolipids, such as OCH [20]; [26], non-glycosidic derivatives of αGalCer [30] and diacylated glycerol based glycolipids derived from Borrelia [32]; [33]. Furthermore, analogs with unsaturated N-acyl chains showed a KRN7000-like cytokine response in human *in vitro* systems (this work and [17]; [20]; [26], while they are Th2-polarizing in mouse [17]. Although not yet investigated in detail, it is possible that these differences could be due to intrinsic differences between mouse and human CD1d proteins. The sequence homology of the antigen binding α1 and α2 domains shows only a moderate level of conservation between the two species, while mouse and human CD1d have distinct ability to present diacylated glycerol based glycolipids derived from Borrelia [33]. It is also particularly notable that the tyrosine-based motif in the human CD1d cytoplasmic tail lacks the ability to bind the cytosolic adaptor protein complex AP-3, which is responsible for the sorting of mouse CD1d into late endosomal and lysosomal compartments [1]; [58]. Based on findings in the current study, we propose that structural analogs of αGalCer with N-linked aromatic branching and weak iNKT cell agonist activity might represent promising candidates for further development as immunomodulatory agents for treatment of autoimmune or inflammatory diseases in humans. Because of the importance of ensuring fine tuning of *in vivo* iNKT cell responses in the treatment of such diseases, such an approach using synthetic glycolipids will need to be evaluated carefully with models that closely approximate the human immune response.

## Supporting Information

Figure S1 Cytokine responses of human iNKT cell clones to CD1d-transfected HeLa cells. CD1d-transfected HeLa cells were used as APC for iNKT cell stimulation in the absence of added glycolipids. After 24h of co-culture, supernatants were harvested, and cytokine levels were measured by ELISA. The sensitivity of detection was 82.3 pg/mL for GM-CSF (white bars), IL-13 (black bars) and IFNγ (hatched bars). The data are shown for a variety of clones indicated at the bottom of the graph. No detectable reactivity was observed with mock-transfected HeLa cells (not shown).
(0.32 MB TIF)

Figure S2 Cytokine responses of a DN iNKT cell clone to a dose range of αGalCer analogs. The data shown were generated with the double negative (DN) HDE3 clone in the same manner as presented in Figure 2 for the CD4+ iNKT cell clone (HDD3).
(0.12 MB TIF)

Figure S3 Cytokine responses of a CD8α+ iNKT cell clone to a dose range of αGalCer analogs. The data shown were generated with the CD8α+ clone HDA7, in the same manner as presented in Figure 2 for the CD4+ iNKT cell clone (HDD3).
(0.12 MB TIF)

Figure S4 Human iNKT cell clone reactivity to OCH and C-Glycoside. (A) CD1d-transfected HeLa cells were incubated overnight with increasing concentrations of KRN7000 (ranging from 0.05 to 100 nM) and OCH or C-Glycoside (ranging from 39 nM to 10 µM) and then used as APC for iNKT cell stimulation. After 24 h of co-culture, supernatants were harvested, and cytokine levels were measured by ELISA. The sensitivity of detection was 27.4 pg/mL for IL-4 and 82.3 pg/mL for IL-13 and IFNγ. The data shown were generated with a CD4+ iNKT cell clone (HDD3), as well as a double negative (DN, HDE3). Dose-response curves are presented for IFNg, IL-4 and IL-13 (top to bottom panels respectively). Mean values measured from triplicate wells are indicated, and standard deviations are shown with brackets. Symbols for each glycolipid are indicated in the legend. (B) The same iNKT cell clones were stained with titrated amounts of analog-loaded hCD1d tetramers (1, 2.5, 5, 10, 20 and 40 nM final concentration), and the measured MFI is plotted for OCH-loaded tetramers (open inverted triangles) and C-Glycoside-loaded tetramers (black diamonds).
(0.13 MB TIF)

Figure S5 Comparison of cytokine responses of human iNKT cell clones between CD1d-transfected HeLa cells and Dendritic Cells, with a limited range of analog concentrations. CD1d-transfected HeLa cells (black bars) and monocyte-derived DCs (white bars) were incubated overnight in parallel with increasing concentrations of KRN7000, C20:2 (tested from left to right at 0.1, 1, 10 and 100 nM,) and higher concentrations of other analogs (10, 100 nM, 1 and 10 µM), then used as APC for iNKT cell stimulation. After 24 h of co-culture, supernatants were harvested, and cytokine levels were measured by ELISA. The sensitivity of detection was 27.4 pg/mL for IL-4 and 82.3 pg/mL for IL-13 and IFNγ. The data shown were generated with a CD4+ iNKT cell clone (HDD3) and a double negative (DN, HDE3).
(0.12 MB TIF)

Figure S6 Dose-dependent cytokine polarization of human iNKT cells with DCs. Variations in cytokine ratios depending on the analog concentration tested, IL-13/IFNγ ratios and IL-4/IFNγ ratios are presented on the bottom and top panel, respectively (calculated as explained in Materials and Methods). Ratios could be calculated only when IL-4 and/or IFNγ were detectable and the # symbol indicates ratios which could not be calculated. Concentrations of antigens are indicated as in Supplementary Figure S5, with low concentrations tested for KRN7000, C20:2 (0.1, 1, 10 and 100 nM) and high concentrations of other analogs (10, 100 nM, 1 and 10 µM). Data presented in this figure were interpreted from results shown in Supplementary Figure S5, and IL-13/IFNγ & IL-4/IFNγ ratios calculated for HDD3 (left panel) and HDE3 (right panel) clones with DCs are presented.
(0.13 MB TIF)

Figure S7 Cytokine responses of human iNKT cell clones to a dose range of aGalCer analogs and costimulation by IL-12/IL-18. CD1d-transfected HeLa cells were incubated overnight with the indicated concentrations of KRN7000 and αGalCer analogs (concentration selected to provide suboptimal stimulation) and then used as APC for iNKT cell stimulation in absence (open bars) or presence of IL-12 and IL-18 (10 ng/mL and 50 ng/mL respectively, black bars). After 24 h of co-culture, supernatants were harvested, and cytokine levels were measured by ELISA. Average cytokine levels and standard deviations are indicated. The sensitivity of detection was 27.4 pg/mL for IL-4 and 82.3 pg/mL for IL-13, GM-CSF and IFNγ. The data shown were generated with a CD4+ iNKT cell clone (HDD3), and similar data (not shown) were obtained with a double negative clone (DN, HDE3).
(0.11 MB TIF)
