# Supplementary figures and images for: α-Galactosylceramide Analogs with Weak Agonist Activity for Human iNKT Cells Define New Candidate Anti-Inflammatory Agents
Source: PLoS One. 2010 Dec 17;5(12):e14374. doi: 10.1371/journal.pone.0014374 (PMC3003687; doi:10.1371/journal.pone.0014374)

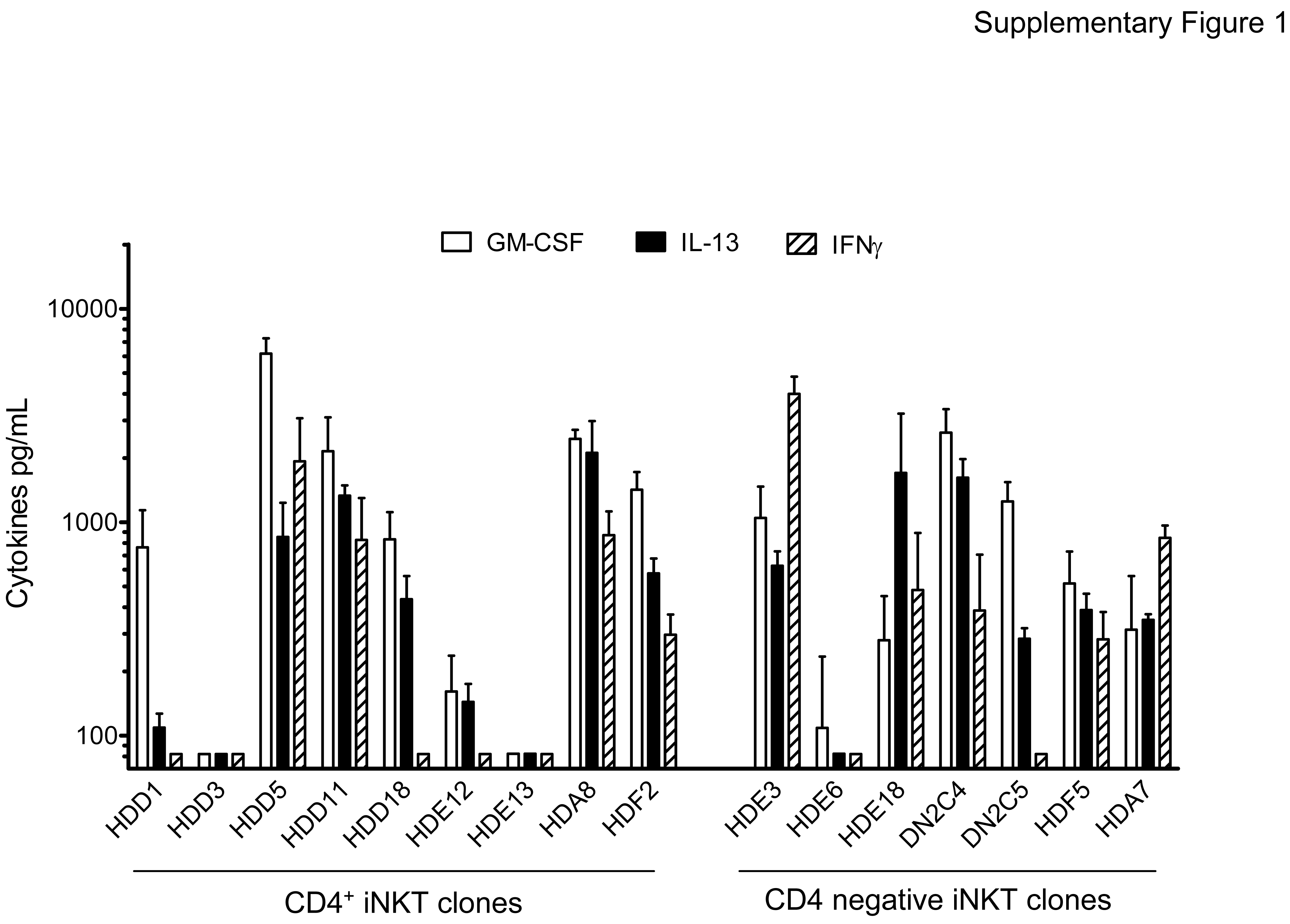

Supplement: Figure S1 — Cytokine responses of human iNKT cell clones to CD1d-transfected HeLa cells. CD1d-transfected HeLa cells were used as APC for iNKT cell stimulation in the absence of added glycolipids. After 24h of co-culture, supernatants were harvested, and cytokine levels were measured by ELISA. The sensitivity of detection was 82.3 pg/mL for GM-CSF (white bars), IL-13 (black bars) and IFNγ (hatched bars). The data are shown for a variety of clones indicated at the bottom of the graph. No detectable reactivity was observed with mock-transfected HeLa cells (not shown). (0.32 MB TIF) [file pone.0014374.s001.tif]

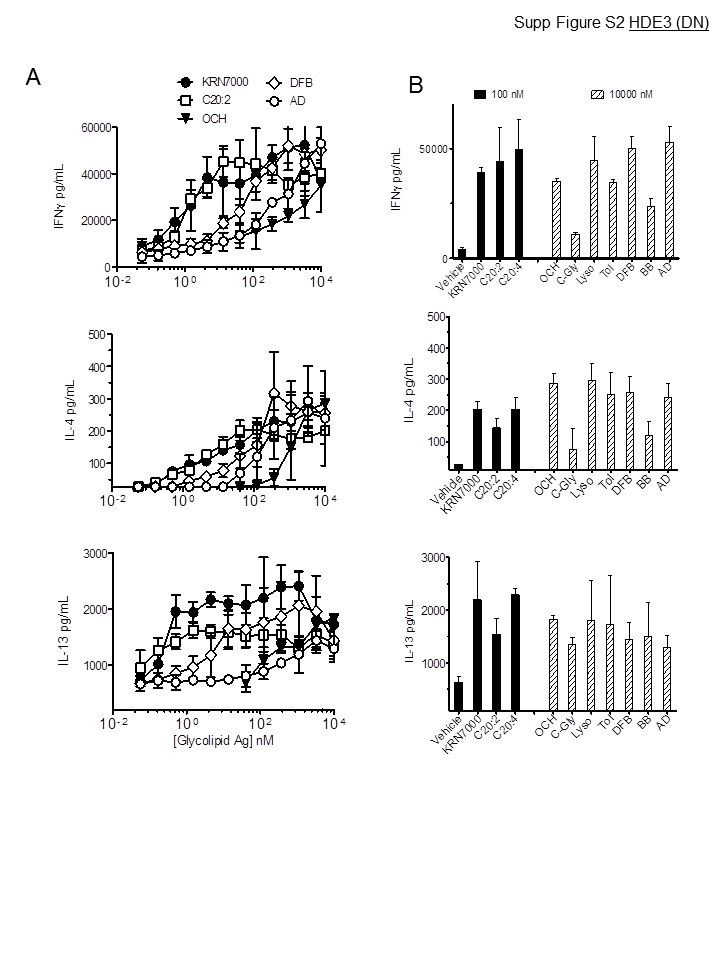

Supplement: Figure S2 — Cytokine responses of a DN iNKT cell clone to a dose range of αGalCer analogs. The data shown were generated with the double negative (DN) HDE3 clone in the same manner as presented in Figure 2 for the CD4+ iNKT cell clone (HDD3). (0.12 MB TIF) [file pone.0014374.s002.tif]

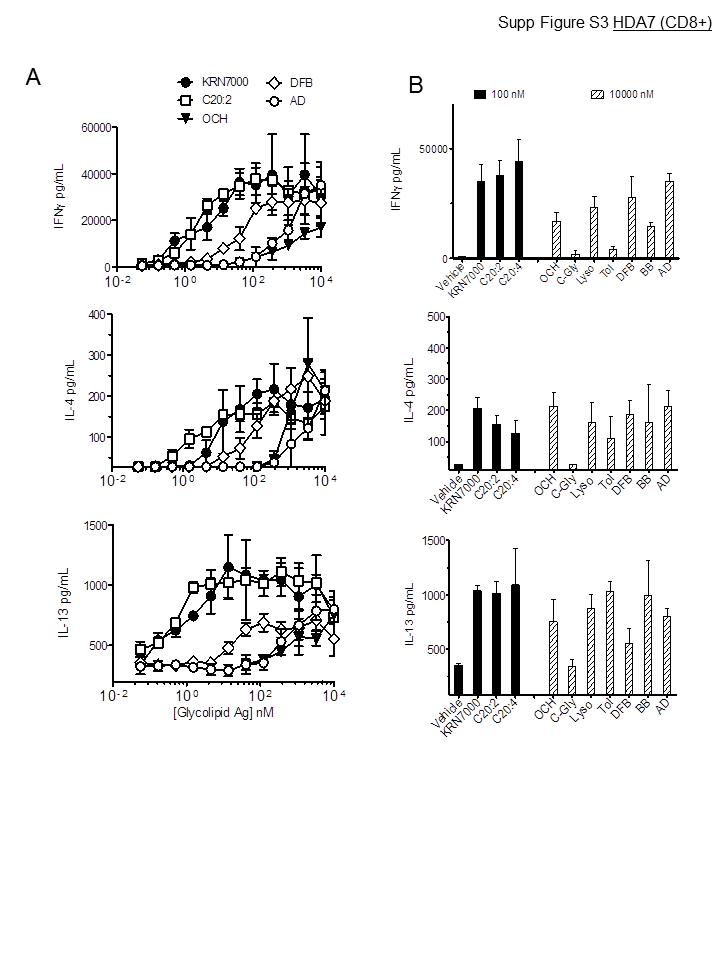

Supplement: Figure S3 — Cytokine responses of a CD8α+ iNKT cell clone to a dose range of αGalCer analogs. The data shown were generated with the CD8α+ clone HDA7, in the same manner as presented in Figure 2 for the CD4+ iNKT cell clone (HDD3). (0.12 MB TIF) [file pone.0014374.s003.tif]

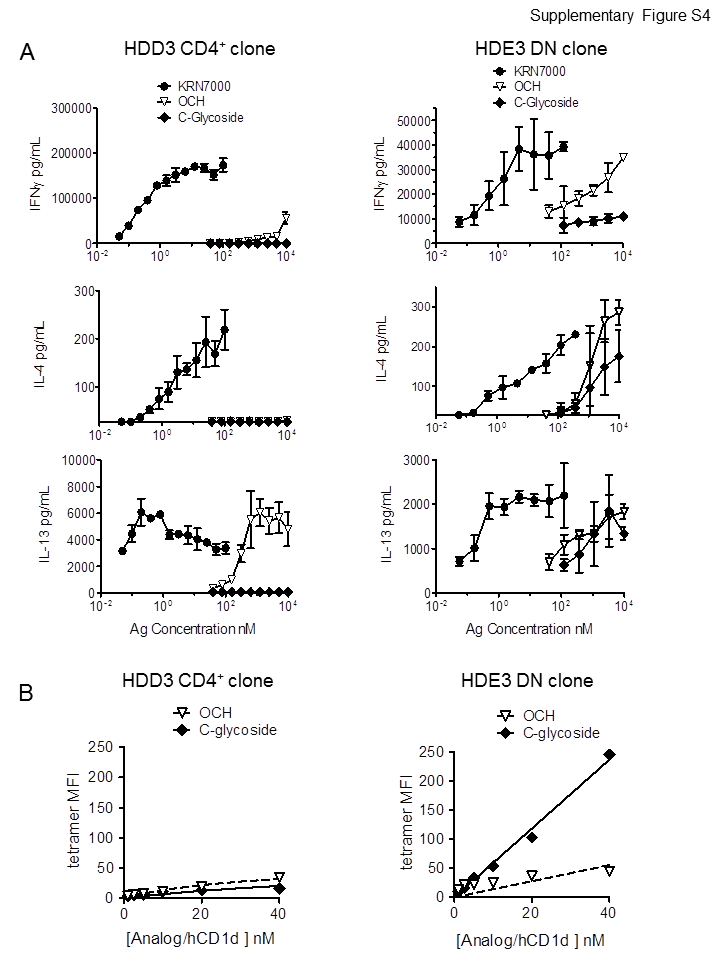

Supplement: Figure S4 — Human iNKT cell clone reactivity to OCH and C-Glycoside. (A) CD1d-transfected HeLa cells were incubated overnight with increasing concentrations of KRN7000 (ranging from 0.05 to 100 nM) and OCH or C-Glycoside (ranging from 39 nM to 10 µM) and then used as APC for iNKT cell stimulation. After 24 h of co-culture, supernatants were harvested, and cytokine levels were measured by ELISA. The sensitivity of detection was 27.4 pg/mL for IL-4 and 82.3 pg/mL for IL-13 and IFNγ. The data shown were generated with a CD4+ iNKT cell clone (HDD3), as well as a double negative (DN, HDE3). Dose-response curves are presented for IFNg, IL-4 and IL-13 (top to bottom panels respectively). Mean values measured from triplicate wells are indicated, and standard deviations are shown with brackets. Symbols for each glycolipid are indicated in the legend. (B) The same iNKT cell clones were stained with titrated amounts of analog-loaded hCD1d tetramers (1, 2.5, 5, 10, 20 and 40 nM final concentration), and the measured MFI is plotted for OCH-loaded tetramers (open inverted triangles) and C-Glycoside-loaded tetramers (black diamonds). (0.13 MB TIF) [file pone.0014374.s004.tif]

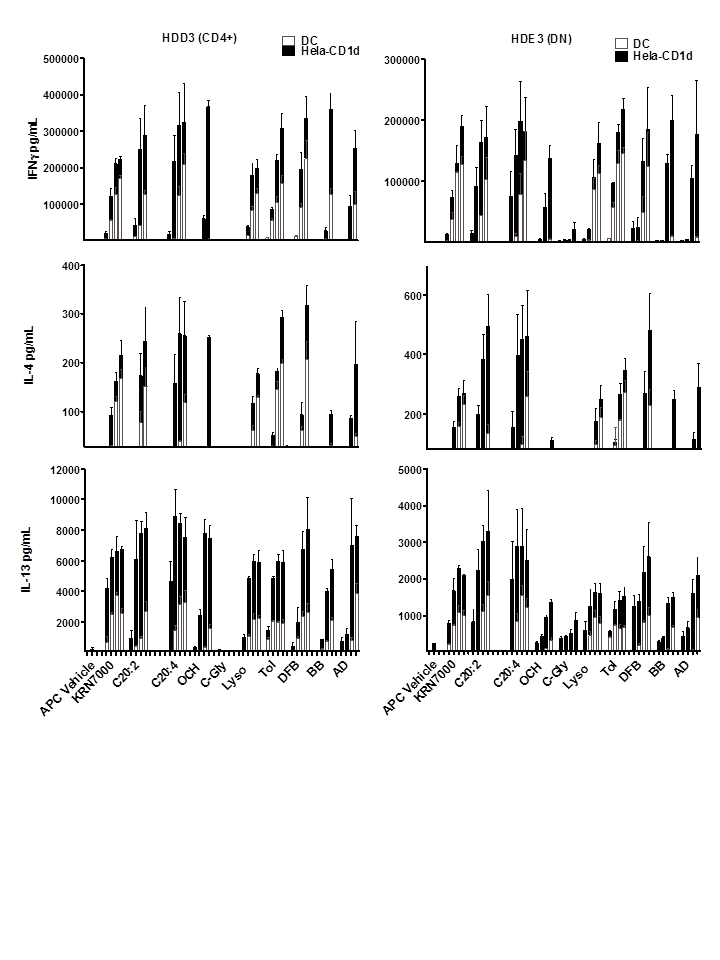

Supplement: Figure S5 — Comparison of cytokine responses of human iNKT cell clones between CD1d-transfected HeLa cells and Dendritic Cells, with a limited range of analog concentrations. CD1d-transfected HeLa cells (black bars) and monocyte-derived DCs (white bars) were incubated overnight in parallel with increasing concentrations of KRN7000, C20:2 (tested from left to right at 0.1, 1, 10 and 100 nM,) and higher concentrations of other analogs (10, 100 nM, 1 and 10 µM), then used as APC for iNKT cell stimulation. After 24 h of co-culture, supernatants were harvested, and cytokine levels were measured by ELISA. The sensitivity of detection was 27.4 pg/mL for IL-4 and 82.3 pg/mL for IL-13 and IFNγ. The data shown were generated with a CD4+ iNKT cell clone (HDD3) and a double negative (DN, HDE3). (0.12 MB TIF) [file pone.0014374.s005.tif]

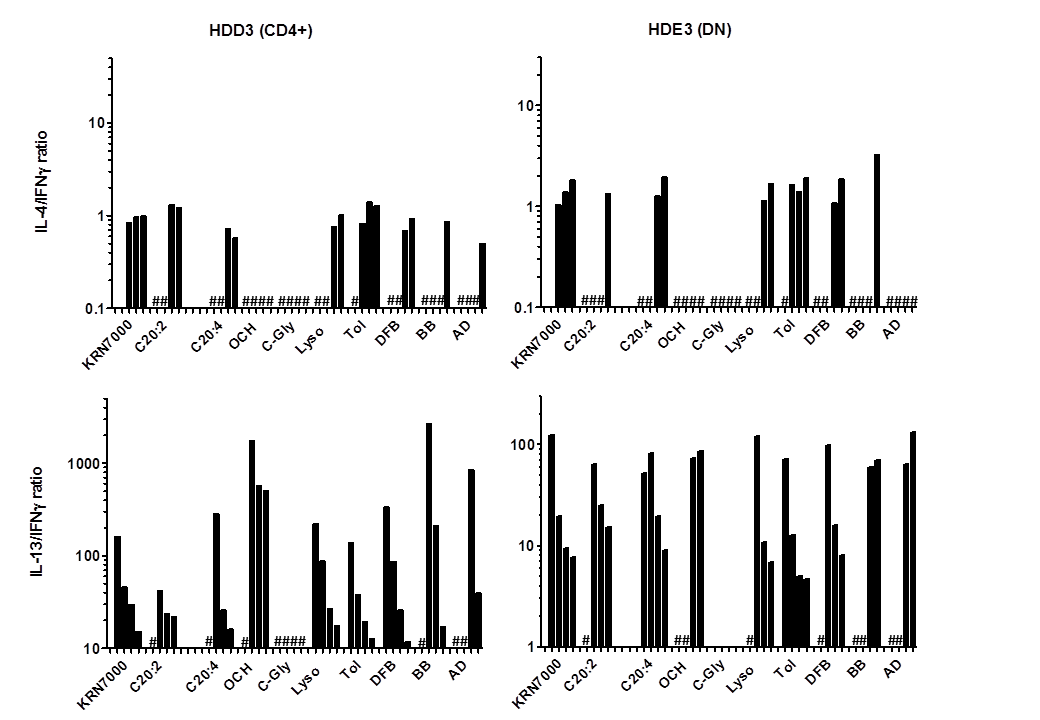

Supplement: Figure S6 — Dose-dependent cytokine polarization of human iNKT cells with DCs. Variations in cytokine ratios depending on the analog concentration tested, IL-13/IFNγ ratios and IL-4/IFNγ ratios are presented on the bottom and top panel, respectively (calculated as explained in Materials and Methods). Ratios could be calculated only when IL-4 and/or IFNγ were detectable and the # symbol indicates ratios which could not be calculated. Concentrations of antigens are indicated as in Supplementary Figure S5, with low concentrations tested for KRN7000, C20:2 (0.1, 1, 10 and 100 nM) and high concentrations of other analogs (10, 100 nM, 1 and 10 µM). Data presented in this figure were interpreted from results shown in Supplementary Figure S5, and IL-13/IFNγ & IL-4/IFNγ ratios calculated for HDD3 (left panel) and HDE3 (right panel) clones with DCs are presented. (0.13 MB TIF) [file pone.0014374.s006.tif]

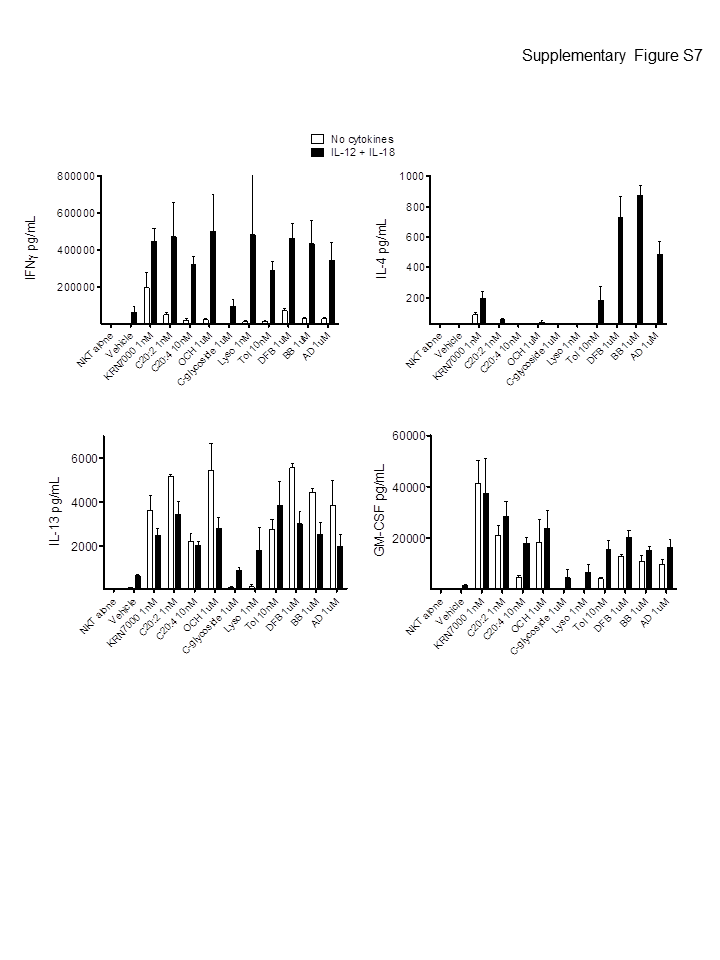

Supplement: Figure S7 — Cytokine responses of human iNKT cell clones to a dose range of aGalCer analogs and costimulation by IL-12/IL-18. CD1d-transfected HeLa cells were incubated overnight with the indicated concentrations of KRN7000 and αGalCer analogs (concentration selected to provide suboptimal stimulation) and then used as APC for iNKT cell stimulation in absence (open bars) or presence of IL-12 and IL-18 (10 ng/mL and 50 ng/mL respectively, black bars). After 24 h of co-culture, supernatants were harvested, and cytokine levels were measured by ELISA. Average cytokine levels and standard deviations are indicated. The sensitivity of detection was 27.4 pg/mL for IL-4 and 82.3 pg/mL for IL-13, GM-CSF and IFNγ. The data shown were generated with a CD4+ iNKT cell clone (HDD3), and similar data (not shown) were obtained with a double negative clone (DN, HDE3). (0.11 MB TIF) [file pone.0014374.s007.tif]
